# Feasibility of establishing acute respiratory infection treatment units (ATU) for improvement of care of children with acute respiratory infection

**DOI:** 10.1186/s12887-022-03240-2

**Published:** 2022-04-08

**Authors:** Aparna Mukherjee, K. R. Jat, Rakesh Lodha, Jagdish Prasad Goyal, Javeed Iqbal Bhatt, Rashmi Ranjan Das, Vinod Ratageri, Bhadresh Vyas, S. K. Kabra, ATU Group, Aparna Mukherjee, K. R. Jat, Rakesh Lodha, Jagdish Prasad Goyal, Javeed Iqbal Bhatt, Rashmi Ranjan Das, Vinod Ratageri, Bhadresh Vyas, S. K. Kabra, Bashir Ahmad Charoo, Daisy Khera, Deepak Singhal, Kuldeep Singh, Partha Sarathi Ray, Samarendra Mahapatro, Prawin Kumar

**Affiliations:** 1 Epidemiology and Communicable Diseases Division, Indian Council of Medical Research, New Delhi, India; 2 Department of Pediatrics, All India Institute of Medical Sciences, New Delhi, 110029 India; 3 Department of Pediatrics, All India Institute of Medical Sciences, Jodhpur, Rajasthan India; 4 Pediatrics, Sher-I-Kashmir Institute of Medical Sciences, Srinagar, Jammu and Kashmir India; 5 Department of Pediatrics, All India Institute of Medical Sciences, Bhubaneswar, India; 6 Department of Pediatrics, Karnataka Institute of Medical Sciences, Hubbali, Karnataka India; 7 Department of Pediatrics, MP Saha Medical College, Jam Nagar, Gujrat India

**Keywords:** Pneumonia, Under five mortality, Upper respiratory infection

## Abstract

**Background:**

Acute respiratory infections (ARI) are the leading cause of morbidity and mortality in children below 5 years of age.

**Methods:**

This multisite prospective observational study was carried out in the Pediatrics’ out-patient departments of 5 medical colleges across India with an objective to assess the feasibility of establishing Acute Respiratory Infection Treatment Unit (ATU) in urban medical college hospitals.

ATU (staffed with a nurse and a medical officer) was established in the out-patient areas at study sites. Children, aged 2–59 months, with cough and/ breathing difficulty for < 14 days were screened by study nurse in the ATU for pneumonia, severe pneumonia or no pneumonia. Diagnosis was verified by study doctor. Children were managed as per the World Health Organization (WHO) guidelines.

The key outcomes were successful establishment of ATUs, antibiotic usage, treatment outcomes.

**Results:**

ATUs were successfully established at the 5 study sites. Of 18,159 under-five children screened, 7026 (39%) children were assessed to have ARI. Using the WHO criteria, 938 were diagnosed as pneumonia (13.4%) and of these, 347 (36.9%) had severe pneumonia. Ambulatory home-based management was done in 6341 (90%) children with ARI; of these, 16 (0.25%) required admission because of non-response or deterioration on follow-up. Case-fatality rate in severe pneumonia was 2%. Nearly 12% of children with ‘no pneumonia’ received antibiotics.

**Conclusions:**

Setting up of ATUs dedicated to management of ARI in children was feasible in urban medical colleges. The observed case fatality, and rate of unnecessary use of antibiotics were lower than that reported in literature.

## Background

Acute respiratory infections (ARI) are the leading cause of morbidity and mortality in children below 5 years of age [1]. Over past few decades, ARI has surpassed acute diarrhea as the leading cause of mortality in under-5 children. Approximately, 808,694 children died from pneumonia in 2017 which amounted to pneumonia killing more than 2500 children every day and accounting for 15% of all deaths of children under the age of five, globally [2]. One of the major issues in dealing with community acquired pneumonia in children is its diagnosis and instituting appropriate management. While the WHO guidelines have facilitated streamlining of the management, there are concerns regarding misclassification of cases, unnecessary use of antimicrobial agents which could contribute to development of resistance to antimicrobial agents, and inadequate treatment of wheezing disorders [3].

Despite the proven benefit of these guidelines [4], there has been some concern about the specificity of the WHO pneumonia algorithm leading to unnecessary use of antibiotics. In a clinical trial of different doses of amoxicillin in the treatment of non-severe pneumonia conducted in Pakistan, children were included by applying WHO criteria and out of 891 cases only 6.8% had radiographic pneumonia [5]. The validity of the WHO guidelines for diagnosing pneumonia in children under 5 years of age was evaluated by several studies in 1990s and the reported sensitivity of WHO criteria ranged between 59 and 81% [6–10].

A critical component of decreasing pneumonia-related mortality is the early identification of patients at risk of treatment failure and the timely provision of supportive care. A designated unit for management of ARI where trained nurses and medical officer are available increases the chance of timely and correct diagnosis of ARI and improved decision making in the management of ARI. Supportive care such as oxygen, salbutamol nebulization can be readily provided. The caregivers can be adequately counselled regarding home-based treatment and the importance of danger signs.

Diarrhea treatment units (DTUs) have played an important role in reducing the mortality due to acute diarrhea in children [11]. On similar lines, we proposed to develop the concept of Acute Respiratory Infection Treatment Units (ATUs) to improve outcomes of ARI in infants and children. A study in another low-middle income country- Mexico had shown that in-service training as well as algorithm-based treatment improved the outcomes of both diarrhea and ARI in under-five children [12]. However, there is lack of information on the feasibility and utility of establishing ATUs.

We carried out this multisite study to assess the feasibility of establishing ATUs which could improve the quality of care of children with acute respiratory infection.

## Methods

### Study design

Multisite prospective cohort study.

### Settings

Urban tertiary care medical college hospitals. Study was carried out at the following five sites across India to represent the geographic variability: 1. Sher-I-Kashmir Institute of Medical Sciences, Srinagar (SKIMS); 2. All India Institute of Medical Sciences (AIIMS), Jodhpur; 3. All India Institute of Medical Sciences (AIIMS), Bhubaneshwar; 4. Karnataka Institute of Medical Sciences (KIMS), Hubbali, Karnataka; and 5. MP Saha Medical College, Jamnagar. Sites were selected based on the following criteria: a. geographical representation of whole country (south, north, east, west), b. prior experience in conducting multisite research, and c. willing and supportive administration. Data were collected at various sites between August 2016 to May 2018.

### Establishing and functioning of ATU

ATUs were established as a part of a research grant. A document describing design of ATUs, its infrastructure, staff, manual for training of staff and working was prepared. Training was provided to the investigators of each site at the coordinating site- AIIMS, Delhi. All recommendations of the document were implemented in five ATUs by the site investigators.

**Fig. 1:**
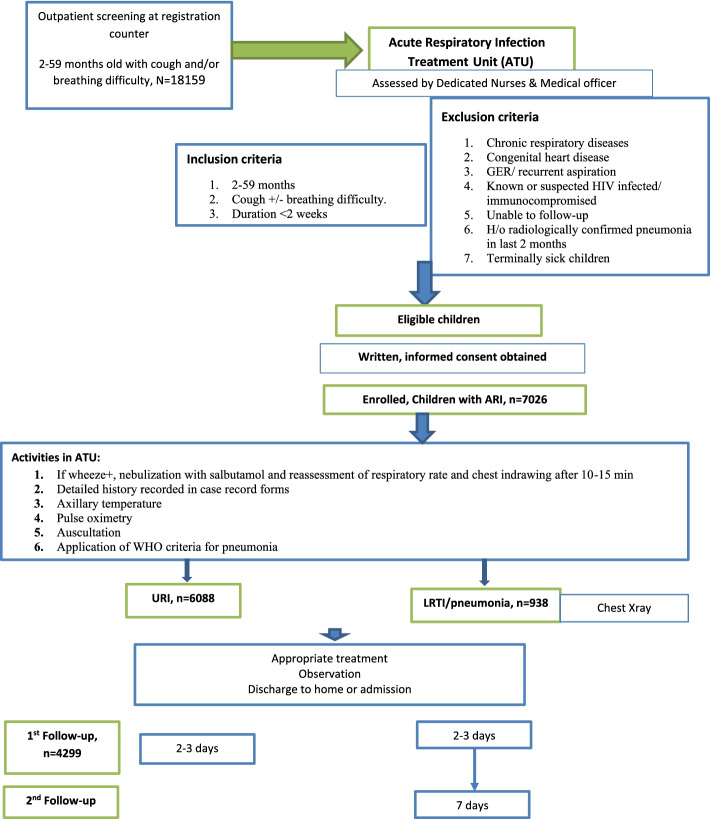
Flow of participants from outpatient counter to final follow-up

ATUs were established at each of the sites within the existing out-patient services in a designated area provided for this purpose. These units offered services during the working hours of the out-patient services. A nurse (with a graduate degree in nursing) and a doctor (minimum qualification being MBBS) served the ATU. The outpatient work-flow was modified to direct the parents/ guardians of the children presenting with cough and/or difficult breathing from the registration counter to the ATU where they were evaluated and managed. In addition, all physicians in OPD and emergency department at study sites were informed about study details and all children between 2 months to 59 months of age with symptoms of ARI were directed to attend ATU during working hours of the out-patient department. All children who presented to ATU were assessed for a history of cough and/or breathing difficulty; trained study staff nurse assessed each child by counting respiratory rate and presence of chest indrawing under supervision of research officer (Fig. 1). Training was provided to the study staff on various aspects of the project such as screening, evaluation, patient management, follow up, and record keeping using a structured module.

The following inclusion and exclusion criteria were used to identify and follow up the group of children with ARI.

### Inclusion criteria

Children, between 2 to 59 months of age with features suggestive of ARI which was defined as the presence of any cough and/or breathing difficulty for duration of less than 2 weeks.

#### Exclusion criteria

Children with any of the following were not enrolled in the study: 1. Patients with chronic respiratory diseases (such as asthma, cystic fibrosis, BPD, airway anomalies), diagnosed in health care facility, 2. Patients with congenital heart disease (suspected based on history of suck-rest-suck cycle and cyanosis)– confirmed by echocardiography or presence of murmur, 3. Patients with GER/ recurrent aspirations (based on history of choking or coughing while feeding or Ba swallow/GER scan), 4. Known or suspected HIV infected/ immunocompromised patient – based on history of recurrent, documented multisite infection or on immunosuppressive therapy, 5. Place of residence outside city where study site is based (as it could influence the ability to follow-up) on the assessment of the site investigator, 6. Unable to attend follow up session, 7. History of radiologically confirmed pneumonia in last 2 months, 8. Terminally sick children- impending respiratory failure, cyanosis at room air, shock [13].

All children who fulfilled case definition of ARI were enrolled in the study after written informed consent from parents/legal guardian.

A detailed clinical history and examination findings of enrolled patient was recorded on a predesigned case record form before any radiological investigation, if required.

#### Clinical pneumonia

The WHO criteria were used for diagnosis of clinical pneumonia [14].

1) cough or difficulty breathing, and

2) age-specific tachypnea (≥50 breaths per minute for children 2–11 months of age and ≥ 40 breaths per minute for children 1–5 years of age) [14].

The severity assessment was also performed as per the WHO criteria [13]. The criteria for severe pneumonia included one of the following: oxygen saturation < 90%, central cyanosis, severe respiratory distress, inability to drink or breastfeed or vomiting everything, altered consciousness, and convulsions [14].

Children with no pneumonia were labelled as having upper respiratory infection (URI).

### Baseline workup

In the ATU, for all patients enrolled in the study, respiratory rate was assessed by the nurse by observing the thorax for 60 s when the child was calm and quiet [14]. Also, the nurse assessed the child for lower chest indrawing by observing the inward movement of the bony structures of lower chest wall. If the child presented with fever and fast breathing, an antipyretic was given and respiratory rate was reassessed at 30 min. If there was audible wheezing and fast breathing, child was given bronchodilator (salbutamol; dose: 0.15 mg/kg) nebulization and respiratory rate was reassessed after 10–15 min. Fever was defined as axillary temperature (recorded using a digital thermometer) ≥37.5 °C, and tachypnea as respiratory rate ≥ 50 breaths/min in children aged 2–11 months and ≥ 40 breaths/min in children from 12 months of age onwards [14]. Oxygen saturation was recorded in all enrolled children with a pulse oximeter. The medical officer auscultated the chest and recorded the findings. In addition, details of symptoms, nutritional and immunization status, previous treatment history, demographic information, anthropometric measurements, clinical examination findings and other relevant details were recorded by the medical officer from research team in ARI case record form especially designed for the purpose. Chest X-ray was done in every 5th child as part of the study. All clinical data were collected without knowledge about the final chest X-ray assessment. For the purpose of the study, all chest X-rays were reported by two investigators using a structured reporting sheet and in case of discordance, a third investigator reviewed the chest X-ray.

All enrolled children treated on OPD basis were followed after 2–3 days. If the children had features of severe pneumonia, they were advised admission. Children diagnosed with lower respiratory infection (LRI) were also followed up at 1 week (± 1 day). Clinical symptoms and findings were recorded in follow-up form. Parents were reminded telephonically 1 day before the scheduled follow-up visit. All in-patients were evaluated in the hospital daily until discharge. If the child did not come for the 7th day’s visit, telephonic contact was established to evaluate the outcome.

### Ethical considerations

The study was performed in accordance with the Declaration of Helsinki. The Institute Ethics Committee of the All India Institute of Medical Sciences, New Delhi (coordinating site) as well the Ethics Committees of the participating institutes *(*1. Sher-I-Kashmir Institute of Medical Sciences, Srinagar (SKIMS); 2. All India Institute of Medical Sciences (AIIMS), Jodhpur; 3. All India Institute of Medical Sciences (AIIMS), Bhubaneshwar; 4. Karnataka Institute of Medical Sciences (KIMS), Hubbali, Karnataka; and 5. MP Saha Medical College, Jamnagar.*)* granted approval to the study protocol*.* The eligible participants were enrolled after receiving informed written consent form the parent/legal guardian. Personal information (paper and electronic registers) was stored in an appropriate manner to ensure full confidentiality.

### Data handling and statistical analysis

A data entry program was developed at AIIMS, New Delhi in MS Access software and sent to all the sites. The data from all study sites were entered locally by study doctor/nurse and sent to AIIMS electronically every fortnightly. Case record forms (CRFs) were sent to AIIMS by post fortnightly and every tenth CRF was cross checked by study nurse/study doctor at AIIMS. Photocopies of the CRFs were retained at the study sites. Discrepancies in data entry was recorded and corrected by contacting respective sites. Each site sent a fortnightly report to AIIMS, New Delhi in a prescribed format.

### Data analysis

Data were analysed using Stata software v.14 (StataCorp., College Station, TX). Descriptive statistics were used to summarize the data. We analysed data for following outcome. 1.Total patients screened, 2. Total patients found eligible during study period, 3. Total excluded with reasons, 4. Total numbers of patients with URI and pneumonia, 5. Total number of patients with severe pneumonia 6. Mortality, lost to follow up and treatment failure. Categorical variables have been expressed as n (%). Continuous variables are summarized a mean (SD) in case of normal distribution, else as median (inter quartile range).

### Quality assurance measures

1. SOPs for screening, evaluation, patient management, follow up, and record keeping were developed, discussed and adopted by all the sites for uniformity.

2. Training of all the study staff (medical officers, nurses, data entry persons) was carried out at respective site by site investigator using SOPs.

3. All centers (except AIIMS, Jodhpur) were visited by investigators to assess the functioning.

3. Skill check of study nurse was done by the medical officer every 3 month.

## Results

A total of 18,159 children were screened in the outpatient departments of the five participating sites, of them 7026 (39%) children had ARI. According to the WHO criteria, 938 (13.4%) and 6088 (86.6%) of the 7026 enrolled children had pneumonia and no pneumonia, respectively; 347 of 938 (36.9%) children had severe pneumonia. The median (IQR) age of the enrolled children was 23 (10, 40) months; 60.5% of them were boys. The demographic and clinical characteristics of the children with ARI are detailed in Table 1.

**Table 1:** Site-wise demographic and clinical characteristics of children with ARI, *n* = 7026

	SKIMS, Srinagar, *N* = 2532	KIMS, Hubbali, *N* = 1802	AIIMS, Bhubaneswar, *N* = 2090	MP Saha Medical College, Jamnagar, *N* = 304	AIIMS, Jodhpur, *N* = 298	Total, *N* = 7026
Age in months	21 (9, 39)	21 (10, 36)	27 (14, 47)	15 (9, 31)	13 (6, 28)	23 (10, 40)
Boys, n (%)	1526 (60.3)	1032 (57.3)	1314 (62.9)	174 (57.2)	205 (68.8)	4251 (60.5)
Weight-for-age, z score	0.33 (−0.41, 1.01)	−1.83 (−2.65, − 1.04)	−0.85 (− 1.75, 0.04)	−1.43 (− 2.42, − 0.58)	−0.98 (− 1.71, − 0.13)	−0.69 (− 1.83, 0.35)
Height/length-for-age, z score	0.6 (− 0.63, 1.95)	−2.79 (−3.95, − 1.62)	−0.69 (− 1.72, 0.52)	−1.21 (− 2.81, 0.39)	−1.15 (− 1.98, − 0.43)	− 0.76 (− 2.36, 0.77)
Weight for height/length, z score	0.02 (−0.61, 0.58)	− 0.3 (− 1.29, 0.56)	−0.75 (− 1.77, 0.32)	−1.13 (− 2.34, 0.24)	−0.55 (− 1.25, 0.36)	−0.29 (− 1.14, 0.53)
Cough, n (%)	2516 (99.4)	1802 (100)	2090 (100)	294 (96.7)	293 (98.3)	6995 (99.6)
Fever, n (%)	1168 (46.1)	1439 (79.9)	873 (41.8)	279 (91.8)	239 (80.2)	3998 (56.9)
Audible wheeze, n (%)	45 (1.8)	1 (0.1)	283 (13.5)	180 (59.2)	3 (1.01)	512 (7.3)
Fast breathing, n (%)	170 (6.7)	168 (9.3)	80 (3.9)	179 (60.7)	118 (39.6)	715 (10.2)
Chest indrawing, n (%)	87 (3.4)	324 (17.9)	46 (2.2)	9 (2.9)	12 (4.03)	478 (6.8)
SpO_2_, mean (SD), in %	96.5 (1.9)	96.3 (3.5)	98.5 (1.9)	97.2 (1.4)	97.2 (2.3)	97.1 (2.6)
Children with SpO_2_ ≤ 92%, n (%)	115 (4.5)	120 (6.6)	10 (0.5)	0 (0)	7 (2.3)	252 (3.6)
Abnormal breath sounds present on auscultation, n (%)	51 (2.0)	345 (19.1)	305 (29.1)	104 (34.2)	76 (25.5)	881 (12.5)

Values are expressed in median (IQR) unless specified

A total of 6341 (90%) children were managed on ambulatory basis while 685 (10%) required hospitalization. Only sixteen (0.25%) children required admission during follow-up, after they were assessed as manageable on ambulatory basis suggesting effective classification of severity of ARI. The compliance to follow-up was 61.2% (4299 children). Table 2 shows information on outcome achieved after treatment of the enrolled children in the five centers. There were some variations in children treated on ambulatory versus hospitalization across the different centers. Approximately 12% of the children who were identified as having no pneumonia as per WHO criteria were administered antibiotics.

**Table 2:** Diagnosis and outcome of patients enrolled in study and managed in ATU, n = 7026

	SKIMS, Srinagar,*N* = 2532	KIMS, Hubbali, *N* = 1802	AIIMS, Bhubaneswar, *N* = 2090	MP Saha Medical College, Jamnagar, *N* = 304	AIIMS, Jodhpur, *N* = 298	Total, *N* = 7026
**No pneumonia/URI**	2347 (92.6)	1410 (78.2)	1917 (91.7)	224 (73.7)	192 (64.3)	6088 (86.6)
**Pneumonia/LRI**	187 (7.4)	392 (21.8)	173 (8.3)	80 (26.3)	106 (35.6)	938 (13.4)
**Severe pneumonia** **(% of pneumonia)**	150 (80.2)	48 (12.2)	36 (20.8)	21 (26.2)	92 (86.8)	347 (36.9)
**Radiologically confirmed pneumonia (in 1273 readable chest Xray)**	170 (36)	319 (52.2)	16 (26.2)	15 (71.4)	66 (61.1)	583 (46)
**Outcome in patients seen on outpatient basis**						
Treated on ambulatory basis	2438 (96.3)	1502 (83.4)	2023 (96.8)	169 (55.6)	209 (70.1)	6341 (90.3)
Admissions	94 (3.7)	300 (16.6)	67 (3.2)	135 (44.4)	89 (29.9)	685 (9.7)
**Outcome in admitted patients**						
Discharged	94 (100)	280 (93.3)	66 (98.5)	120 (88.9)	86 (96.6)	646 (94.3)
Left against medical advice	-	19 (6.4)	-	13 (9.6)	-	32 (4.7)
Death	-	1 (0.3)	1 (1.5)	2 (1.5)	3 (3.4)	7 (1.0)
**Admission after being sent home**	7 (0.3)	4 (0.3)	1 (0.05)	2 (1.2)	–	16 (0.25)
**Proportion of patients with no pneumonia (WHO) who received antibiotics**	394/2345 (16.8%)	84/1410 (5.9%)	136/1917 (7.1%)	99/224 (44.2%)	30/192 (15.6%)	743/6088 (12.2%)

All values are expressed as n (%), diagnosis of no pneumonia, pneumonia and severe pneumonia was based on the WHO criteria [14]

### Mortality

Among the study subjects, 7 children died [case fatality 0.01%]. Case fatality rate among children with pneumonia was 0.75% and among severe pneumonia was 2%. Details of children who died are given in Table 3. All were below 18 months (4 were infants) of age. Three children had weight-for-length < − 2 Z score. X ray film of chest was obtained in 4; one patient had consolidation, while in three no significant abnormalities were present. Procalcitonin (PCT) was performed only in one child who had consolidation on CXR and PCT was 0.53 ng/mL (more than cut off value of 0.5 ng/mL). Three children were assessed to have no pneumonia in beginning but subsequently died of severe bronchiolitis. Cause of death was assessed as sepsis in one child, while 6 children died of severe bronchiolitis or viral infection associated wheezing. Only one patient had severe hypoxemia at time of presentation.

**Table 3:** Details of the enrolled children who died (*n* = 7), all were hospitalized

Sl No	Age	Gender	Diagnosis (WHO)	WHZ	CXR finding	SpO_2_ (%)	PCT level (ng/mL)	Treatment (whether antibiotic received)	Final Diagnosis
1	15 mo	F	Pneumonia	−2.32	Consolidation	84	0.53	Required mechanical ventilation and antibiotics	Sepsis with septic shock with right side empyema
2	6 mo	F	No pneumonia	–	No CXR done	96		Received antibiotics	WALRI with FTT
3	13 mo	M	No pneumonia	−1.44	No significant abnormality on CXR	96		Required mechanical ventilationDid not receive antibiotics	WALRI 1st Episode
4	8 mo	M	No pneumonia	−3.3	No CXR done	98		Received antibiotics	Severe Bronchiolitis
5	3 mo	F	Severe Pneumonia	−2.75	No significant abnormality on CXR	100		Did not receive antibiotics	Acute bronchiolitis
6	1 mo	M	Severe Pneumonia	1.74	No significant abnormality on CXR	100		Required non-invasive ventilation, Did not receive antibiotics	Acute bronchiolitis
7	18 mo	M	Severe Pneumonia	−0.33	No CXR done	100		Received antibiotics	Acute bronchiolitis

*WHZ* Weight for height/length Z score, *CXR* Chest X ray, *PCT* procalcitonin, *WALRI* wheeze associated lower respiratory tract infection, *FTT* failure to thrive

## Discussion

In this multi-center observational study across five sites in India, a total of 18,159 children below 5 years of age attending outpatient departments of urban medical college hospitals were screened; 7026 children were assessed to have acute respiratory infection who were managed in the ATUs. A total of 6341 (90%) children were managed on ambulatory basis while 685 (10%) required hospitalization. Only 16 (0.25%) children managed on ambulatory basis, required admission when they did not respond or deteriorated on follow up.

There are multiple reports describing epidemiology of ARI from different parts of India. These include hospital based as well as community-based studies. The methodology followed in these studies varied. A systematic review published in the recent past suggests that total burden of ARI reported in literature from developing countries varies between 20 and 40% in hospital-based studies [15]. The proportion of children with ARI enrolled in our study (39%) is consistent with the reported literature [16, 17]. A recent review suggests that about 10% of all ARI may be pneumonia [17]. In the present study, 13% of all ARI were diagnosed as pneumonia, possibly due to the setting of the study being tertiary care hospitals. Proportion of children with ARI having severe pneumonia is variable and varies from 10 to 20% [18, 19]. Severe pneumonia was diagnosed in 36.9% of children with pneumonia in our study. We used the revised definition of WHO where severe and very severe pneumonia have been clubbed together. Additionally, two sites (Jamnagar and AIIMS Jodhpur) enrolled more pneumonia and severe pneumonia that increased the overall number of severe pneumonias. The main reason for the variation may be differences in the spectrum of illness of children seeking care at different centers; this is also seen in the numbers of children presenting with wheezing. Jodhpur site enrolled only in last 6 months of project while Jamnagar enrolled very small number of children due to local logistics.

We did not do baseline statistics to document impact of ATU on outcome of management of ARI. This is one of the limitations of the study. However, in present study success rate for diagnosis, case fatality and use of antibiotics were significantly better as compared to historical documentation in literature from similar setting [20–22]. In the present study, we enrolled, prescribed treatment, did follow up and recorded outcome in 90% of our patients. Majority (90%) of the children were managed on ambulatory basis, only 10% required hospitalization. Only 16 (0.25%) children who were initially on ambulatory treatment required admission. A study from Pakistan comparing ambulatory with in-patient management for non-severe pneumonia reported hospitalization in the ambulatory management arm ranging from 1.7–2.8% [23]. Only 12% of the children with URTI were treated with antibiotics. This proportion varied between centres ranging from 44.2 to 5.9%. Mortality of hospitalized children varies between 10 and 50% and is dependent on the type of hospital. Case fatality rate amongst severe pneumonia cases in our study was only 2%. This could possibly be because of focused approached to appropriate diagnosis of pneumonia along with adequate management in terms of hospital admission and antibiotic usage along with aggressive follow-up. Exclusion of children with underlying diseases/ co-morbid conditions would have also contributed to lower case fatality. The concept of dedicated ATU facilitated the decision making and implementation in both diagnosis and management of ARI in children. Also, our observations suggest that possibly viral infections causing severe ALRI were responsible for mortality in younger children below 18 months of age.

A recent report suggests that almost 75% of patients with upper respiratory infection were getting antibiotics [24]. As majority of URI are due to viral infections, antibiotics are not required. Unnecessary antibiotic prescription is one of the major factors for acquired drug resistance. Encouragingly in our study, only 12% of children with no pneumonia received antibiotics.

This study is a preliminary endeavor to evaluate the feasibility and impact of acute respiratory infection treatment units in improving care of children with acute respiratory infection. Results suggest that it is feasible to develop ATUs and the outcomes are encouraging. Our deduction is based on comparison of data from literature. We did not collect baseline data from individual sites to objectively document impact of ATU. Reasons for not collecting data before intervention were multiple: duration of project was 24 months and ARI data may be affected according to season, we did not have enough time to collect 12 months of data before and after intervention.

Our study was carried out in medical college hospitals, data on the proportion of children treated in such hospitals are not available. However, children with ARI in our study as well as other studies are around 40%, therefore such units in hospitals with large inflow of patients including medical colleges may be useful. Good outcome of children treated in ATUs may be because of standardized case management, aggressive follow-up and telephonic contact. We suggest carrying out randomized controlled trials comparing outcomes in ATUs vs. non-ATU centers for assessing the impact of ATUs. Also, operational studies to assess generalizability and further studies of longer duration may be carried out involving multiple centers with high infant and under-five mortality with documentation of pre- and post- intervention data.

## Conclusion

The study results suggest that ATUs can be established within existing out-patient departments. The assessment by nurse and doctor for ambulatory treatment or hospitalization worked very well. While there was no control group/ period, results suggest that units could effectively manage children with ARI; 90% of children being managed on ambulatory basis, and antibiotic usage was also low. The study also provides epidemiological data about magnitude of problem of ARI, information regarding the clinical aspects of pneumonia, and outcome of under-five children in public health care facilities.

## Data Availability

The datasets generated and analysed during the current study are not publicly available as the study team is carrying out further analyses of the same but are available from the corresponding author on reasonable request.
